# A Complaint of a Criticism

**Published:** 1910-05-14

**Authors:** C. Elsee

**Affiliations:** Chairman of the Board. Rugby


### Institutional Letter-Box.

### A COMPLAINT OF A CRITICISM.

*To the Editor of* The Hospital.

Sir,—The Board of Management of the Hospital of St. Cross have seen with much surprise and regret the report published in The Hospital of April 30—surprise at its general tone, and regret that it will tend to throw obstacles in the way of their endeavours to make the work of the hospital efficient. No appreciation is shown of the generosity of the gentleman who, in his lifetime, founded and liberally endowed the hospital, and who made it a chief point in his building to secure for the patients not only care and comfort but also brightness and pleasant outlook while confined to their beds; but harsh criticism only—not always quite just—is directed at his benefaction. I may mention that even the name of the founder is not given correctly, and the position of the wards wrongly described. An unjustifiable doubt again is thrown on the household management for the patients, which, while economical, is excellent. The Board are well aware of imperfections in the hospital, as, for instance, in the accommodation for the nursing staff, as compared with that generally provided at the present day, and they are anxious to remedy them; but they feel that a report written in the apparent spirit of this one will only hinder their efforts, so far successful, in keeping up a good and efficient staff, and so ensuring the best treatment for the patients. Is it not at least unusual for an unofficial visit and inspection to be made and a report published without the courtesy of a communication with the Board of Management?—Yours truly,

C. Elsee,
Chairman of the Board.

Rugby, May 7.

[We publish Mr. Elsee's letter with pleasure, as it shows that the authorities of this hospital are alive to the importance of keeping their institution up-to-date and efficient. With the complaints made in the letter we need deal very briefly. By a regrettable oversight the founder's name was printed "Woods" instead of "Wood": we fail to see in what way the plan of the wards is wrongly described. Full credit was given in the concluding sentence of the first paragraph for the merits of the institution and for the endeavour to keep it in good order. Unofficial visits and inspections are daily paid to many hospitals by those interested in hospital work without any preliminary communication with the Board of Management, and such reports as we have published would largely lose their value were they submitted to the authorities of the hospitals concerned for sub-editing before publication. We hardly think it necessary to point out that Sir Henry Burdett's reports on British hospitals are written in no antagonistic or carping spirit, but with the sole intention of stimulating interest in these institutions. They are impartial expressions of opinion by one who is a recognised expert in the subject with which he deals, and any criticisms they contain must therefore be calculated to move the authorities to look over their institution and find out for themselves how far the blame, where such is given, is merited, and in what manner existing defects may be remedied. It is not always necessary expressly to show appreciation of the generosity of hospital founders: the comfort of the patients and the thorough efficiency of the institution are the best expressions of such appreciation. In conclusion, we may assure Mr. Elsee that the report will not tend to throw obstacles in the way of endeavours to promote efficiency, but rather act as a stimulus to the public to assist the committee in these endeavours.—Ed. The Hospital.]

### TIGHT NECK-CLOTHING.

*To the Editor of* The Hospital.

